# A Rare Presentation of Sclerosing-Type Rhabdomyosarcoma in an Underserved Patient: A Case Report

**DOI:** 10.7759/cureus.82110

**Published:** 2025-04-11

**Authors:** Lorena Rivera, Delaram Pourkalbassi, Desiree Boisson, Nick Taylor, Ernest Metelus, Ammaar Jan, Oudhay S Sohal, Damian Casadesus

**Affiliations:** 1 Internal Medicine, St. George’s University School of Medicine, Miami, USA; 2 Internal Medicine, Jackson Memorial Hospital, Miami, USA; 3 Internal Medicine, Ross University School of Medicine, Miami, USA; 4 Internal Medicine, American University of the Caribbean School of Medicine, Miami, USA

**Keywords:** adult rhabdomyosarcoma, health disparities, metastatic rhabdomyosarcoma, sclerosing rhabdomyosarcoma, underserved patient population, vai chemotherapy

## Abstract

A man in his early 40s, with no previous medical history, presented to the emergency room with right leg swelling. It had started more than one month prior to the presentation, but the patient had not sought medical attention due to lack of insurance. He underwent an ultrasound, computed tomography (CT) scan, and magnetic resonance imaging (MRI). The studies revealed a mass in the right vastus medialis muscle. A biopsy revealed a high-grade rhabdomyosarcoma (RMS) with sclerosing features. The patient started treatment with vincristine, actinomycin D, and ifosfamide (VAI). He continued chemotherapy as an outpatient, but was lost to follow-up. He returned four months later with metastatic disease.

## Introduction

Rhabdomyosarcoma (RMS) is a malignant mesenchymal tumor composed of primitive skeletal muscle cells (rhabdomyoblasts) that have not undergone complete differentiation. RMS is the most common type of soft tissue sarcoma in children. Roughly half of all pediatric patients diagnosed with soft tissue sarcoma have RMS [1]. Soft tissue sarcomas account for approximately 7% of all cancers in children and only 1% of cancers in adults [2]. Therefore, progressive RMS in an adult is somewhat rare and warrants further study.

This report presents an adult man diagnosed with RMS who had no significant medical history. RMS in adults most commonly arises in the extremities [3], as was also the case in the patient presented in this study. However, it can emerge in nearly all anatomic sites of the body. Other common sites of involvement include the trunk, genitourinary tract, and head and neck [3]. The four major histological types of RMS include embryonal, alveolar, pleomorphic, and sclerosing subtypes. The patient described in this report was diagnosed with sclerosing rhabdomyosarcoma (SCRMS). His condition was further complicated by metastasis to the brain. Metastasis occurs in approximately 15% of RMS patients. Unfortunately, metastatic cases are generally associated with a poor prognosis and low survival rate. We will discuss the multimodal approaches used in this patient’s diagnosis and management. By presenting this case, we aim to raise awareness about the aggressive nature of RMS in adults and highlight the significant impact that health disparities and lack of proper follow-up can have on prognosis.

## Case presentation

A man in his 40s, with no significant prior medical history, presented to the Emergency Department with right leg swelling and discomfort that had persisted for one month following a work-related injury. On examination, his vital signs were stable: blood pressure 121/79 mmHg, temperature 36.7°C, heart rate 68 bpm, respiratory rate 18 breaths per minute, and oxygen saturation 100% on room air. The patient was not in acute distress. Examination of the right lower extremity revealed a 14-15 cm tender mass on the medial aspect of the thigh, accompanied by 1+ pitting edema. Cardiopulmonary and abdominal examinations were normal. He was alert, awake, and oriented, with a normal neurological examination. Relevant laboratory results on admission were unremarkable (Table 1).

**Table 1:** Relevant laboratory values on admission with reference values.

Laboratory Test	Patient Laboratory Value	Normal Laboratory Range
White blood cells	8,200/mm^3^	4,500-11,000/mm^3^
Hemoglobin	14.3 g/dL	13.5-17.5 g/dL
Hematocrit	40.9%	41-53%
Platelets	254,000/mm^3^	150,000-400,000/mm^3^
Potassium	3.9 mEq/L	3.5-5.0 mEq/L
Prothrombin time	12.7 seconds	11-15 seconds
Partial thromboplastin time	29 seconds	25-40 seconds
International normalized ratio	0.94	<1.1
Red blood cells	4.46 million/mm^3^	4.3-5.9 million/mm^3^
Mean corpuscular volume	91.7 mm^3^	80-100 mm^3^

**Figure 1:**
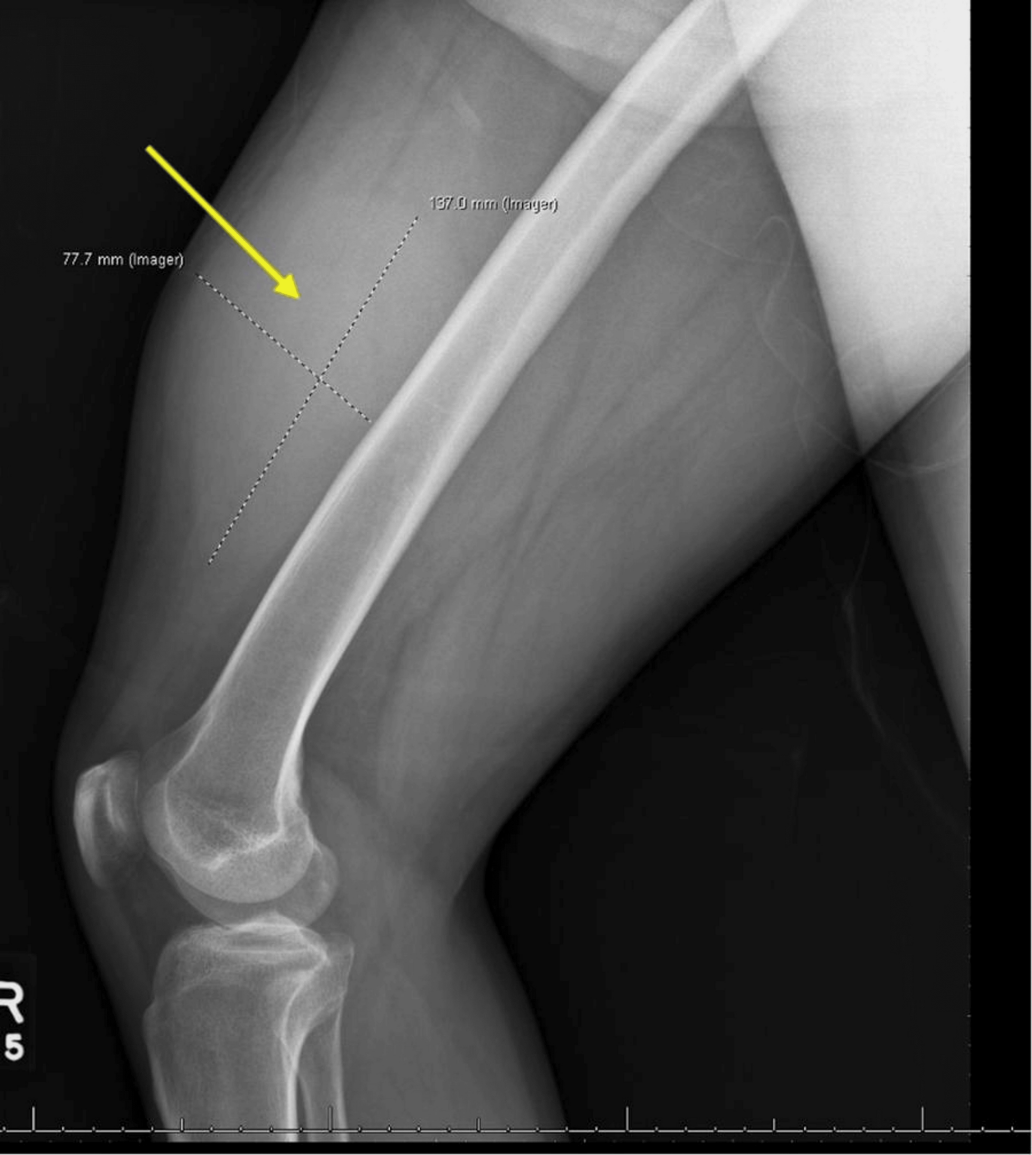
X-ray of the right femur revealed a 77.7 x 137 mm mass (arrow) and ruled out a femoral fracture.

The ultrasound of the leg suggested a hematoma, while an X-ray of the femur revealed a 77.7 × 137 mm mass and ruled out a femoral fracture (Figure 1). A computed tomography (CT) scan raised concerns for a neoplastic process, with liposarcoma as a possible diagnosis. A magnetic resonance imaging (MRI) revealed a large 15 cm mass in the right vastus medialis muscle, featuring areas of hemorrhage, necrosis, and a solid enhancing component. A biopsy confirmed high-grade RMS with sclerosing features, measuring 15.5 cm. The C/A/P CT at that time showed no metastatic disease in the abdomen or pelvis but noted mildly increased pulmonary nodules, the largest being 4 mm. A peripherally inserted central catheter line was placed, and chemotherapy with vincristine, actinomycin D, and ifosfamide (VAI) was initiated by the Oncology team. The patient completed one cycle of VAI but subsequently lost follow-up due to financial difficulties and lack of insurance.

**Figure 2:**
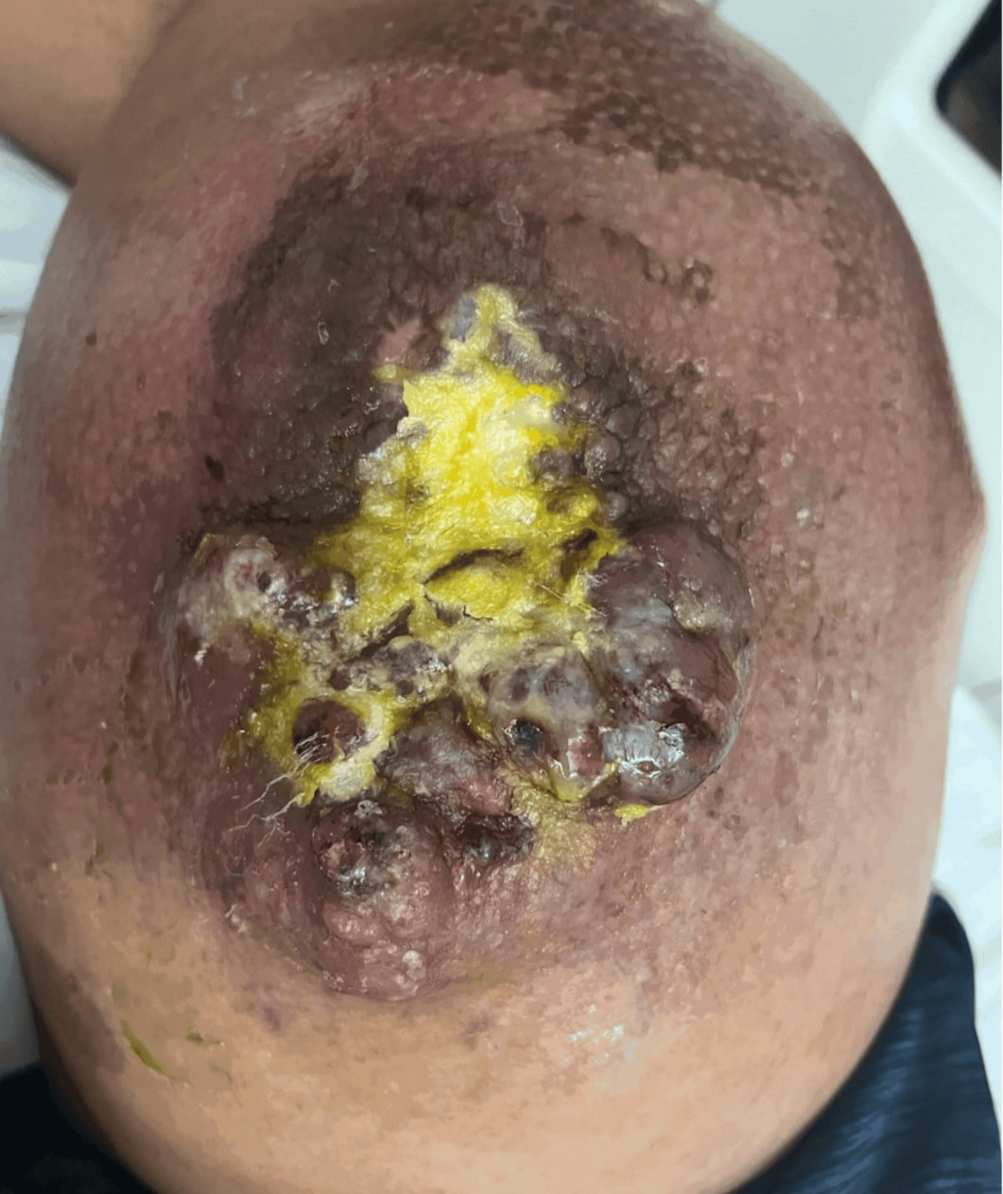
Right thigh multilobulated mass.

Four months later, he returned to the Emergency Department with worsening edema. Upon physical examination, there was an increase in the size of the leg mass. The MRI of the lower extremity indicated progressive disease, with the mass increasing in size and encasing the deep femoral vessels and saphenous nerve, involving the femoral diaphysis with mild cortical scalloping but no bony invasion. The patient started an urgent second cycle of VAI. The chest CT revealed numerous new noncalcified nodules consistent with intrathoracic metastatic disease. Given these findings, the patient was informed that further treatment would no longer be curative. He underwent a total of four cycles of VAI, managing the treatment well until he presented again to the Emergency Department with acute blurred vision, leg pain, and persistent left-sided headaches seven months after the initial diagnosis. Examination of the leg indicated a multilobulated mass in the right lower extremity with surrounding edema (Figure 2). The patient was in significant pain in the right lower extremity; however, no speech difficulties, motor weakness, personality changes, or other neurological symptoms were observed.

**Figure 3:**
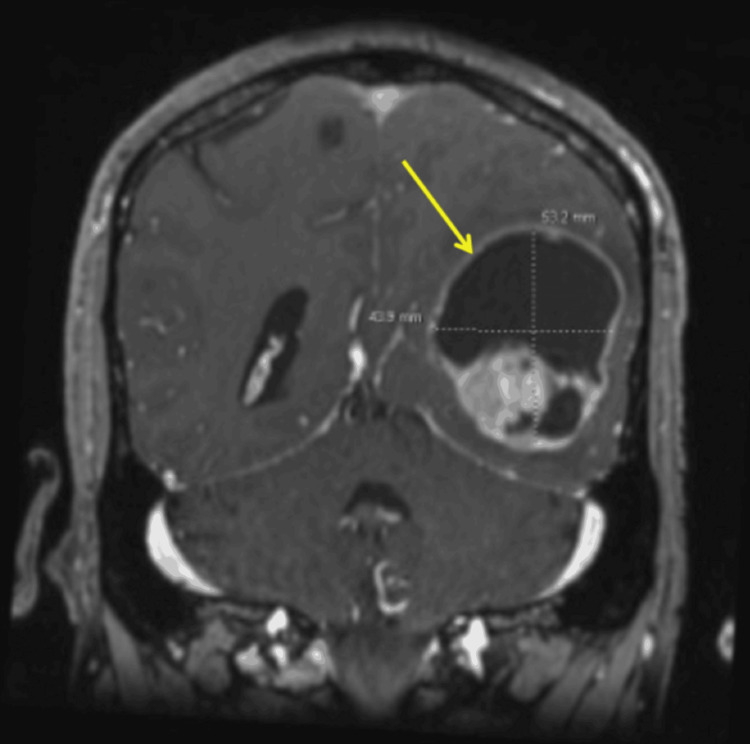
MRI of the brain revealed an enhancing, irregular mass with a large cystic component (5.3 x 4.3 cm, arrow) in the left parietotemporal lobe. MRI, magnetic resonance imaging

**Figure 4:**
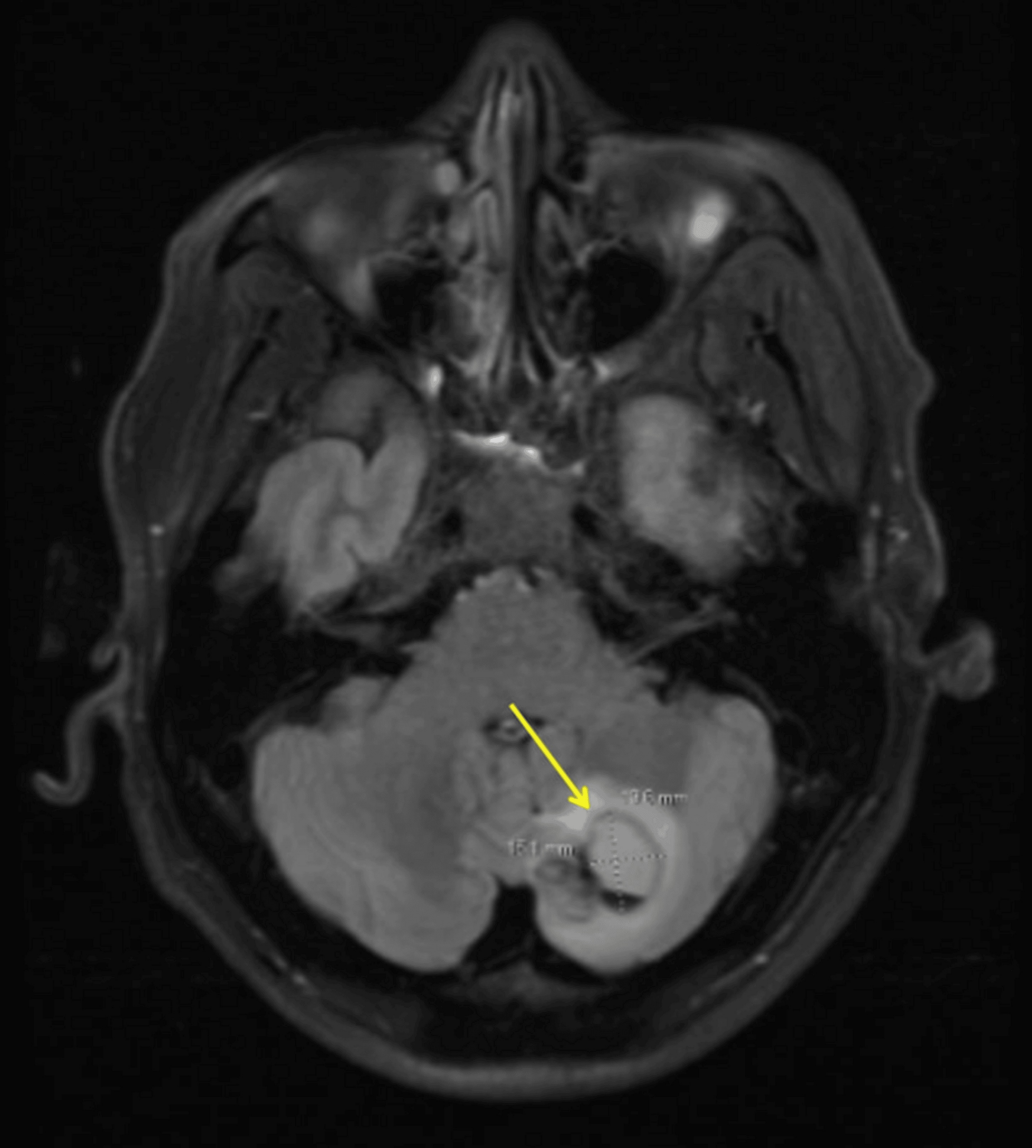
MRI of the brain revealed enhancing lesion in the left cerebellum (1.9 x 1.5 cm, arrow), suggestive of metastatic lesions. MRI, magnetic resonance imaging

**Figure 5:**
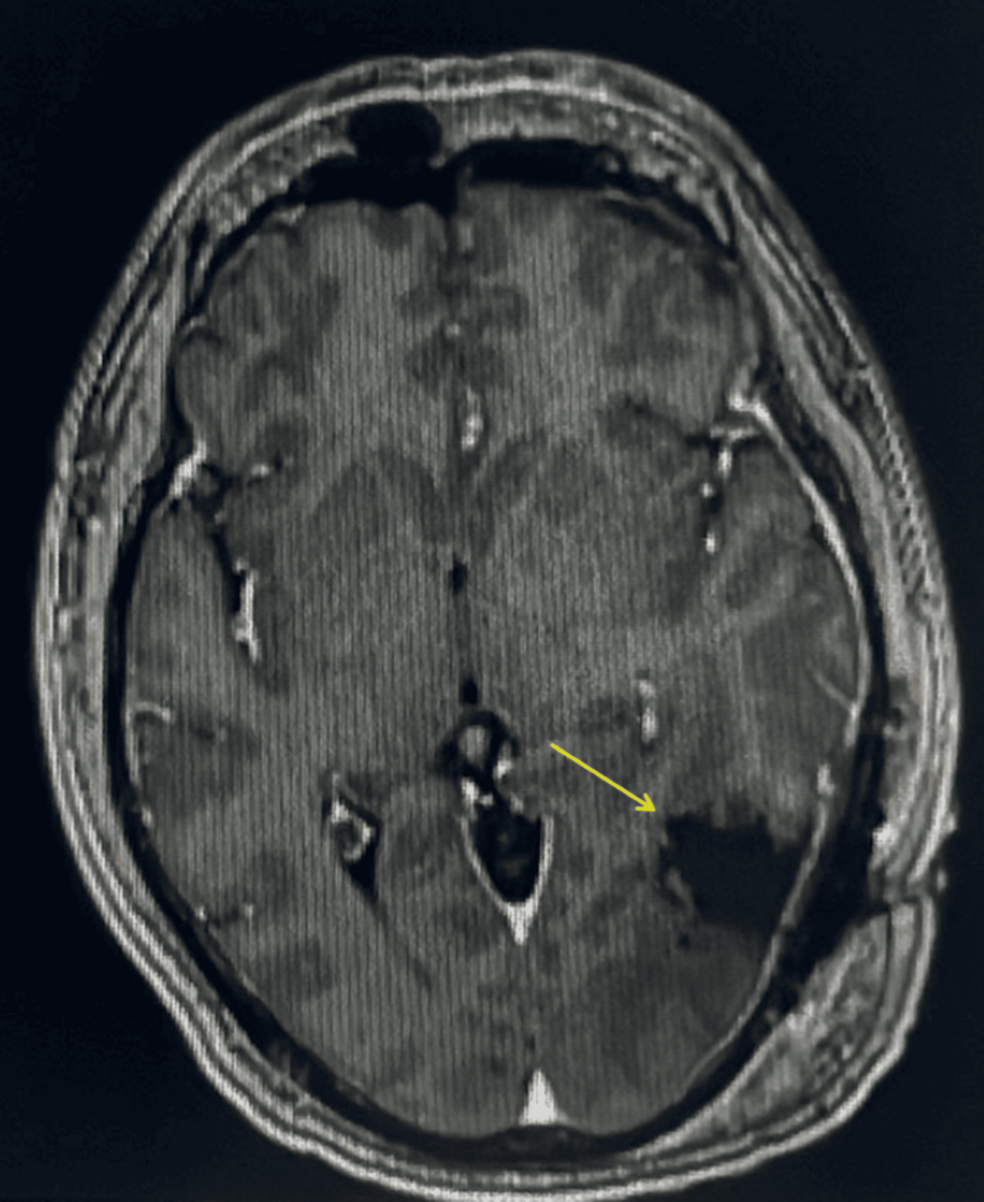
Post-operative brain MRI revealed an interval left temporal craniotomy (arrow) with a post-surgical cavity and no suspicious enhancement in the surgical area. MRI, magnetic resonance imaging

An MRI of the brain was performed in the emergency room because of his presentation with acute blurred vision and persistent left-sided headaches, with a seven-month history of SCRMS. The MRI results revealed an enhancing, irregular mass with a large cystic component (5.3 × 4.3 cm) in the left parietotemporal lobe (Figure 3), and an additional enhancing lesion in the left cerebellum (1.9 × 1.5 cm), suggestive of metastatic lesions in the dominant hemisphere (Figure 4). The patient subsequently underwent a left temporal-occipital-parietal craniotomy for the removal of the supratentorial tumor and mass decompression. The post-operative physical examination indicated that the patient was awake, alert, and oriented to person, place, and time. The patient started treatment with dexamethasone for two weeks and was prescribed Keppra 500 mg twice daily. The post-operative MRI revealed an interval left temporal craniotomy with a post-surgical cavity and no suspicious enhancement in the surgical area (Figure 5). The patient was discharged with close follow-up by the Medical Oncology team.

## Discussion

This report presents a case of a man in his early 40s who was diagnosed with rapidly progressing RMS in his right vastus medialis muscle. RMS is a rare malignant tumor that originates in soft or connective tissues, or bones, and is classified as a type of sarcoma. It is typically more prevalent in the pediatric population, with only 1%-2% of cases occurring in adults [4]. In the U.S., approximately 400 cases are diagnosed annually. The survival rate for children with RMS ranges from 70% to 90%, whereas adults generally have a less favorable prognosis, with a five-year survival rate of approximately 20% [5,6].

RMS is categorized into four main histological types: embryonal, alveolar, sclerosing/spindle cell, and pleomorphic. Embryonal RMS is the most common subtype and has the best prognosis, while alveolar and pleomorphic RMSs are associated with poorer outcomes [7]. In this case, the patient was diagnosed with SCRMS, a rare subtype that accounts for 5%-10% of all RMS cases [8]. SCRMS was recently designated as an independent pathological category, distinct from embryonal RMS, due to its unique morphological features. It can manifest in various body locations, such as the head and neck, extremities, and trunk. The primary symptom is typically a lump or swelling in the soft tissue beneath the skin, often in the head and neck region, and usually painless.

Unlike pediatric cases, SCRMS in adults tends to follow a more aggressive course. Approximately 15% of RMS patients present with metastatic disease, which correlates with a poor prognosis and limited improvement in survival rates over time. Common metastatic sites include the lungs, liver, lymph nodes, and bone, with the lungs being the most frequent site of metastasis. Lung-only metastases are seen in 18%-24% of cases and generally have better outcomes compared to metastases in other locations [9]. Brain metastases are much rarer, occurring in only 1%-8% of cases and typically developing late in the disease progression [10]. In our case, lung metastasis was observed five months after the initial diagnosis, followed by rapid brain metastasis to the left parietotemporal lobe, left cerebellum, and the inferior part of the left temporal lobe, approximately eight months after the diagnosis of the primary tumor. Our patient presented with persistent severe pain in the right lower extremity and demonstrated an aggressive course due to its rapid progression and metastatic spread. 

The standard treatment for RMS includes a combination chemotherapy regimen of VAC/VAI (vincristine, actinomycin D, and cyclophosphamide/ifosfamide), along with radiation therapy and surgical tumor excision. This regimen significantly enhances outcomes for localized RMS cases. However, there has been limited progress in improving outcomes for metastatic RMS patients. Recent studies suggest that incorporating low-dose maintenance chemotherapy, specifically low-dose vinorelbine and cyclophosphamide, following standard treatment, can enhance outcomes for RMS patients. Maintenance therapy has been associated with a five-year disease-free survival rate of 78%, compared to 70% for those not receiving it. Furthermore, the five-year overall survival rate was 87% for patients on maintenance therapy, versus 74% for those without it [11].

Despite advances in treating RMS, challenges like low cure rates, drug resistance, and tumor recurrence continue to be major issues. Additionally, socioeconomic factors, including income, employment status, education, race/ethnicity, insurance coverage, and family support, can significantly impact a patient’s access to timely medical care, often leading to more advanced stages of cancer at diagnosis.

## Conclusions

This report aims to highlight the multifactorial components involved in approaching, diagnosing, and managing high-grade RMS with sclerosing features in an adult with no past medical history. However, more significantly, this case underscores the undeniable impact of various multidisciplinary factors on this patient’s prognosis. These factors include, but are not limited to, health disparities such as healthcare and financial access, lack of insurance, and the importance of timely and diligent follow-up. In addition, this patient’s sixth-grade education level may have led to a lack of medical knowledge, which reminds us how vital our patients' full understanding of their respective conditions and follow-up is.

Additionally, this report of an underserved patient highlights the substantial effect of health disparities regarding the promptness of clinical management and follow-up for this presentation of SCRMS. Despite advancements in treatment modalities, including surgical intervention and chemotherapy/radiotherapy, the prognosis remains poor due to rapid progression and metastasis to the brain and lungs. The improvement of prognosis is contingent upon utilizing molecularly targeted agents, such as ferroptosis agents. Although new treatment options have been established, socioeconomic factors and equal access to healthcare play a vital role in determining clinical outcomes for patients, as highlighted in this case report. In summation, it is essential to address and establish a multidisciplinary approach to the socioeconomic and medical challenges faced by patients with rare and aggressive malignancies, particularly those who are indubitably underserved.
